# Cell-Free DNA Methylation as Blood-Based Biomarkers for Pancreatic Adenocarcinoma—A Literature Update

**DOI:** 10.3390/epigenomes5020008

**Published:** 2021-04-09

**Authors:** Stine Dam Henriksen, Ole Thorlacius-Ussing

**Affiliations:** 1 Department of Gastrointestinal Surgery, Aalborg University Hospital, 9000 Aalborg, Denmark; otu@rn.dk; 2 Clinical Cancer Research Center, Aalborg University Hospital, 9000 Aalborg, Denmark

**Keywords:** pancreatic cancer, pancreatic ductal adenocarcinoma, epigenetic, DNA methylation, diagnostic biomarker, prognostic biomarker, cell-free DNA

## Abstract

Pancreatic adenocarcinoma has a horrible prognosis, which is partly due to difficulties in diagnosing the disease in an early stage. Additional blood-born biomarkers for pancreatic adenocarcinoma are needed. Epigenetic modifications, as changes in DNA methylation, is a fundamental part of carcinogenesis. The aim of this paper is to do an update on cell-free DNA methylation as blood-based biomarkers for pancreatic adenocarcinoma. The current literature including our studies clearly indicates that cell-free DNA methylation has the potential as blood-based diagnostic and prognostic biomarkers for pancreatic adenocarcinoma. However, still no clinical applicable biomarker for pancreatic adenocarcinoma based on DNA methylation do exist. Further well-designed validation studies are needed.

## 1. Introduction

Pancreatic adenocarcinoma (PDAC) is the fourth leading cause of cancer death with a five-year survival rate less than 10% [1]. A crucial reason for the poor prognosis is that many patients are diagnosed with late-stage disease, which unfortunately makes curative treatment impossible.

Actually, the only clinical available biomarker for PDAC is carbohydrate antigen-19-9 (CA-19-9). CA-19-9 has prognostic properties, as elevated levels are more common in advanced cancer stages. Further, preoperative increased level of CA-19-9 is associated with decreased survival and low resectability rate [2,3]. Unfortunately, CA-19-9 lacks sufficient sensitivity and specificity for use as a diagnostic marker [4,5]. In addition, 10% of the population lacks the ability to produce CA-19-9 due to Lea-b-blood group status [2,4,6].

It would be a major advance for patients if additional blood-borne biomarkers were available to facilitate the detection of PDAC at an early stage. A blood-based diagnostic marker for PDAC would be ideal for screening high-risk individuals or even in patients with an intermediate risk of developing PDAC, such as patients with chronic pancreatitis, late-onset diabetes and familiar disposition to PDAC [7]. Such biomarkers could serve as a supplement to existing clinical tools in the diagnostic work-up of patients suspected of PDAC. Furthermore, additional prognostic biomarkers would be highly beneficial by facilitating the initial identification of patients with more aggressive tumor biology, optimize therapeutic decision making and promote individualized therapy.

The objective of this present paper is to give an update on the current literature on cell-free DNA methylation as blood-based biomarkers for PDAC.

## 2. The Importance of Blood-Based Biomarkers for PDAC

Blood-based markers have several advantages over tissue-based markers. The current standard of care for diagnosing PDAC involves examination of tumor tissue either by fine needle aspiration cytology or histological examination of biopsies or surgical specimens [8], which all are invasive procedures entailing a risk of complications. Blood-based tests are minimally invasive, involving only limited discomfort, and have no major complications. They can easily be repeated to enable close monitoring of the disease to evaluate response to treatment or early detection of recurrence [9,10,11].

As pancreas, or at least part of it, is difficult to access for any kind of tissue sampling, it calls for much easier methods as liquid biopsies. In addition, the size of the tumor may limit the ability to sample tissue adequately, and tissue biopsies may not be an accurate representation of the tumor due to intra-tumor heterogeneity [11,12,13]. There can also be molecular differences between the primary tumor and metastatic lesions, and thus a tissue biopsy from the primary lesion most likely will not represent the metastatic lesions [12]. In cases where tumor tissue specimens are unavailable from either the primary tumor or the metastatic lesions, blood-based markers may represent an alternative or a supplement to existing tools used in the diagnostic work-up and treatment of PDAC [11,12,13].

## 3. Cell-Free DNA

The presence of cell-free DNA in peripheral blood has been known for decades [14]. Cell-free DNA in the serum of patients with cancer was first described in 1977, where it was shown that patients with cancer had a larger amount of cell-free DNA than healthy individuals [15]. In 1983, similar results were described for pancreatic disease: PDAC patients had significantly higher levels of cell-free DNA compared to patients with chronic or acute pancreatitis [16].

The biology of cell-free DNA remains to some extent unclear. However, the release of nucleic acids into the blood is thought to be related to the apoptosis and necrosis of cancer cells or secretion by cancer cells [17]. Furthermore, it has been suggested that a part of the cell-free DNA may act as micrometastases or originate from circulating tumor cells undergoing cell death [17].

Tumors are usually heterogenic, with a mixture of different cancer cell clones and normal cell types, resulting in the release of both tumor-derived and wild-type cell-free DNA into the blood during tumor progression [14,17]. It is still challenging to differentiate circulating tumor DNA from circulating non-tumor DNA. This challenge is enhanced by the fact that several benign conditions also are associated with an increased level of cell-free DNA due to the shedding of nucleic acids into the blood by apoptotic and necrotic cells [18,19]. In recent years, free circulating or cell-free DNA have become of major interest as tools for minimal invasive diagnostics, i.e., “liquid biopsy”. It is an alternative approach to cancer tissue biopsy for analyzing genetic and epigenetic aberrations.

## 4. DNA Methylation

DNA methylation consists of the addition of a methyl (CH3) residue to a cytosine preceding a guanosine, known as a CpG dinucleotide [20,21,22]. CpG dinucleotides are in CpG-rich regions known as CpG islands, where the majority of methylated CpG dinucleotides are in repetitive intragenomic sequences. Furthermore, 60% of genes in the human genome contain one or more CpG islands in the promoter region. However, only 5% of these promoter sequences are methylated under normal conditions [20,21,22]. Methylated DNA results in a tightly packed chromatin structure, and unmethylated DNA is associated with lightly packed chromatin [20]. Aberrant DNA methylation (hypo- and hypermethylation) is a fundamental part of carcinogenesis. Global DNA hypomethylation of repetitive sequences is a part of early carcinogenesis and may lead to chromosomal instability. DNA hypermethylation often occurs in the CpG islands of the promoter sequences of genes. Hypermethylation in the promoter regions of tumor suppressor genes result in downregulation/silencing of tumor suppressor function, whereas hypomethylation in promoter regions of oncogenes may result in increased gene expression [20,21].

In PDAC aberrant methylation has been shown in precursor lesion of the pancreas [23,24]. A progressive increase in the prevalence of methylation has been demonstrated with increasing dysplasia and it has been suggested that specific genes can be target for aberrant methylation at different stages of pancreatic neoplastic progression [23,24,25].

## 5. Cell-Free DNA Methylation as Diagnostic Biomarkers for PDAC

Melnikov et al. (2009) was the first to publish a study on plasma DNA methylation changes as blood-based diagnostic biomarkers of PDAC. Using microarray analysis, the promoter region of five genes were detected as hypomethylated in cell-free DNA of PDAC patients (*n* = 30), reaching a 76% sensitivity and 59% specificity [26].

Liggett et al. (2010) used the same microarray analysis to identify seventeen gene promoter regions aberrantly methylated in plasma cell-free DNA when comparing patients with PDAC (*n* = 30), chronic pancreatitis (*n* = 30) and healthy individuals (*n* = 30). The gene panel reached a 90.8% sensitivity and a 91.2% specificity for PDAC when compared to chronic pancreatitis. In addition, a 78% sensitivity and a 81.7% specificity was demonstrated when differentiating patients with chronic pancreatitis and healthy individuals [27]. However, no further validation of the results has been published.

Park et al. examined in 2012 by methylation specific PCR promoter hypermethylation of a six gene panel (*NPTX2*, *UCHL1*, *SARP2*, *ppENK*, *p16* and *RASSF1A*) in plasma cell-free DNA of PDAC patients (*n* = 16), patients with chronic pancreatitis (*n* = 13), and healthy individuals (*n* = 29). Hypermethylation of all six genes were found both in plasma from patients with PDAC and patients with chronic pancreatitis, however the frequency was higher in cancer patients, although not statistically significant [28]. Afterwards, a study including 104 PDAC patients, 60 chronic pancreatitis patients, and five patients with benign gallstone disease focused on the methylation status of *NPTX2* [29]. *NPTX2* hypermethylation was found in pretreatment plasma from 84% of PDAC patients, 33% of patients with chronic pancreatitis and none of the patients with benign gallstone disease (*p*-value = 0.016).

The methylation status of a panel of five tumor suppressor genes (*p16*, *p14*, *RASSF1A*, *APC* and *DCC*) was evaluated by Kawasaki et al. in 2013 [30]. PDAC patients (*n* = 47) were compared to patients with other types of cancer (*n* = 197). Pretreatment plasma was analyzed by methylation specific PCR. Hypermethylation of *p16* and *RASSF1A* was detected in 17% and 34% of patients with PDAC, respectively. Similar methylation frequency of *p16* and *RASSF1A* was seen for patients with several other types of cancer. Except for hepatocellular carcinoma where 73.3% of patients had hypermethylated *RASSF1A*. Hypermethylation of *APC*, *DCC* and *p14* were also detected in PDAC patients, however less frequent [30].

In 2013 Joo Mi Yi et al. analyzed cell-free DNA promoter hypermethylation of *BNC1* and *ADAMTS1* in pretreatment serum from patients with PDAC (*n* = 42) and healthy volunteers (*n* = 26) [31]. The sensitivity was 79% and 48% for *BNC1* and *ADAMTS1*, respectively. Specificity was 89% for *BNC1* and 92% for *ADAMTS1*. In addition, *BNC1* and *ADAMTS1* showed a promising sensitivity of 90% for stage I PDAC [31]. The results were validated in 39 patients with PDAC and 95 age-matched controls, and the combination of *ADAMTS1* and *BNC1* showed a convincing AUC of 0.95 (0.91–0.98) [32]. The panel was also analyzed in patients with chronic pancreatitis (*n* = 8). Unfortunately, seven of the eight patients with chronic pancreatitis were hypermethylated in either of the two genes, indicating the test fail to differentiate PDAC and chronic pancreatitis [32].

A recent study by Nidhi Singh et al. [33] examined methylation status of four genes (*SPARC*, *UCHL1*, *PENK* and *NPTX2*). Cell-free DNA in plasma of patients with PDAC (*n* = 61), patients with chronic pancreatitis (*n* = 22) and healthy controls (*n* = 21) were analyzed. To differentiate PDAC and chronic pancreatitis *SPARC* reached an AUC of 0.76 (0.65–0.87) and a similar performance was seen for early stage PDAC., whereas the other genes did not reach statistical significance between PDAC and chronic pancreatitis. In general, the methylation level of all four genes was significantly higher in PDAC patients compared with the healthy controls [33].

In 2015 primarily based on a literature review [34], which addressed genes aberrantly methylated in PDAC, a panel of 28 promoter regions was designed by our group and examined in both a diagnostic and prognostic biomarker study [35]. The aim was to evaluate the overall performance of genes previously examined separately as markers for PDAC. Cell-free DNA promoter hypermethylation in plasma was analyzed in a large cohort of PDAC patients (*n* = 95). A clinically relevant control groups of patients with chronic pancreatitis (*n* = 97), patient screened for but not having upper gastrointestinal cancer (*n* = 27) and patients with acute pancreatitis (*n* = 59) were included [35]. Based on a subgroup of eight genes from the panel a diagnostic prediction model (age > 65 years, *BMP3*, *RASSF1A*, *BNC1*, *MESTv2*, *TFPI2*, *APC*, *SFRP1* and *SFRP2*) for PDAC was developed reaching an AUC of 0.86 (95% CI 0.81–0.91). Very importantly, the prediction model was independent of cancer stage. Subsequently, an external validation study of the diagnostic gene panel has been initiated, and in addition the additive effect of CA-19-9 was analyzed. So far, the results are promising, especially as the test is developed using patients with benign pancreatic disease as the control group, which enables differentiation of cancer specific hypermethylation and hypermethylation in response to an inflammatory pancreatic reaction, as observed in chronic or acute pancreatitis. This aspect is important because chronic pancreatitis is a significant risk factor for PDAC and the differentiation of patients with PDAC from patients with chronic pancreatitis is a known clinical challenge.

Recently other studies combining methylation analyses with other diagnostic modalities has been published. Fujimoto et al. analyzed methylation status of *RUNX3* in plasma cell-free DNA in combination with CA-19-9 in patients with PDAC (*n* = 55), chronic pancreatitis (*n* = 12) and healthy volunteers (*n* = 80). *RUNX3* methylation had a sensitivity of 50.9% for PDAC, with a 93.5% specificity using the total control group. By combining *RUNX3* methylation and CA-19-9 the sensitivity increased substantial (85.5%) [36]. Furthermore, Keiko Shinjo and coworkers [37] examined methylation status of five genes (*ADAMTS1*, *HOXA1*, *PCDH10*, *SEMA5A* and *SPSB4* in serum from patients with PDAC (*n* = 47) and healthy controls (*n* = 14). The mean methylation level of the five genes in cell-free DNA was not significant different between cancer patients and controls. However, twenty-three PDAC patients had at least one of the genes methylated leading to a 49% sensitivity and an 86% specificity. Additionally, *KRAS* mutation in cell-free DNA was detected in twenty-three PDAC patients. When combining *KRAS* mutation and methylation status the diagnostic performance improved (68% sensitivity and 86% specificity) [37]. Both studies lack validation, which is needed to confirm the results.

Using various methods, which is outside the scope for this present review, the methylation status of a broad spectrum of genes has been analyzed in cell-free DNA in order to find genes with potential to work as diagnostic biomarkers for PDAC. Table 1 summarizes the above-mentioned diagnostic studies. Hypermethylation of several genes has been detected in plasma and serum from patients with PDAC. At low frequency hypermethylation of the same genes are also present in plasma and serum from patients with chronic pancreatitis and healthy controls [27,28,32,35]. In conclusion, cell-free DNA hypermethylation has the potential to work as diagnostic biomarkers for PDAC. However, none of the genes jet examined has the potential to function as an individual diagnostic marker and the performance of the previously described gene panels do not allow any of them to be used as a stand-alone test for PDAC diagnosis. Nevertheless, so far, the results from the validation study of the diagnostic gene panel developed by our group, which is based on hypermethylation of eight genes in combination with CA-19-9 shows a promising performance. This together with the other studies illustrating improved performance in combination with *KRAS* mutation [37] or CA-19-9 [36], indicate a panel of hypermethylated genes in combination with other diagnostic modalities might be needed to bolster the diagnostic power, which seems reasonable keeping in mind the molecular heterogenicity of PDAC.

The studies of DNA methylation have brought us closer to a diagnostic biomarker for PDAC, but we are not quite there yet. Many studies are of small sample size and still lack validation. According to the five-phase structure of biomarker development described by Pepe et al. [38] studies on DNA methylation as diagnostic biomarkers for PDAC only reach phase 2 (see Table 1). Currently, no diagnostic biomarker for PDAC based on cell-free DNA methylation has reach phase 3, which involves a retrospective longitudinal study of clinical specimens collected from cancer patients before their clinical diagnosis. In addition, no phase 4 studies (prospective studies) or phase 5 studies, which estimate the reduction of cancer mortality afforded by the biomarker, do exist. In general, further research is needed. Well-designed validation studies, including both patients with benign and malignant pancreatic disease (etc. patients with late-onset diabetes and individuals with a familiar disposition to PDAC) testing broad gene-panels in combination with other diagnostic modalities will hopefully get closer to a clinical useful diagnostic marker.

## 6. Cell-Free DNA Methylation as Prognostic Biomarkers for PDAC

As mentioned earlier a progressive increase in aberrant DNA methylation, both hyper- and hypomethylation, has been demonstrated with increasing dysplasia [23,24], indicating there to be a prognostic potential in DNA methylation changes.

In general, only very few studies on the prognostic value of hypermethylated cell-free DNA in PDAC do exist. In 2017 our group investigated cell-free DNA in PDAC regard to stage classification and survival [25,39].

### 6.1. Number of Hypermethylated Genes According to Cancer Stage and Survival

Similar to other studies, a study by our group demonstrated that hypermethylated cell-free DNA is detectable in all stages of PDAC [25,35,39]. Furthermore, the study discovered that patients with distant metastases had an even higher number of hypermethylated genes in cell-free DNA compared with patients with localized disease [25]. Distant metastasis has previously been reported to result in a larger amount of cell-free DNA [10]. However, the level of cell-free DNA was not associated with cancer stage in our study [25]. The study of Park et al. [28] was not able to show similar association between metastatic PDAC and a higher number of hypermethylated genes in cell-free DNA, which could be due to a lack of power or differences in genes analyzed. However, our results on cell-free DNA are consistent with a study on PDAC tumor tissue [23]. These observations suggest that hypermethylated promoter regions accumulate during the course of PDAC development and progression.

Another study by our group demonstrated that the number of hypermethylated genes in cell-free DNA was associated with survival. Patients with more than ten hypermethylated genes were more likely to die during the first year after diagnosis than patients with fewer hypermethylated genes [39]. Similar association between number of hypermethylated genes and poor survival has been reported for head and neck cancer [40].

### 6.2. Methylated Genes Associated with Distant Metastasis

Cell-free DNA hypermethylation of seven individual genes *ALX4*, *BNC1*, *HIC1*, *SEPT9v2*, *SST*, *TFPI2,* and *TAC1* has been associated with distant metastasis in a study by our group [25]. Likewise, *HIC1* hypermethylation in tumor tissue has previously been detected in PDAC stage III-IV [41]. In addition, Park et al. described cell-free DNA hypermethylation of *NPTX2* to increase significantly with aggravating tumor stage [28]. Nidhi Singh and coworkers [33] also found cell-free DNA hypermethylation of *NPTX2* and *SPARC* to be more pronounced in stage IV PDAC. Similar prognostic value of *NPTX2* was not found in our study.

### 6.3. Methylated Genes According to Cancer Stage

To enable differentiation of PDAC patients according to cancer stage two prediction models were developed by our group [25]. A panel based on the hypermethylation status of eight genes (*SEPT9v2*, *SST*, *ALX4*, *CDKN2B*, *HIC1*, *MLH1*, *NEUROG1*, and *BNC1*) enabled with high performance (AUC of 0.87) the distinction of stage IV PDAC patients from PDAC patients with stage I-III. Another panel (*MLH1*, *SEPT9v2*, *BNC1*, *ALX4*, *CDKN2B*, *NEUROG1*, *WNT5A*, and *TFPI2*) enabled the differentiation of potentially resectable disease (stage I and II) from non-resectable disease (stage III and IV) (AUC of 0.82) [25].

### 6.4. Methylated Genes According to Survival

Furthermore, the association between cell-free DNA hypermethylation and survival of PDAC patients was investigated by our group [39]. Overall, promoter hypermethylation had a negative impact on survival. However, hypermethylation of a few specific genes had a positive effect on survival. Hypermethylation of *SFRP1*, *BMP3*, and *TFPI2* was significantly associated with impaired survival in stage IV disease [39]. In concordance with our findings, hypermethylation of *SFRP1* in tumor tissue has been identified as an independent risk factor for poor overall survival in breast cancer [42] and renal cancer [43]. In addition, hypermethylation of *TFPI2* in hepatocellular carcinoma tumor tissue is associated with advanced cancer stage and shorter survival [44], similar to our results for cell-free DNA in PDAC.

Based on hypermethylated cell-free DNA prediction models for survival of patients with PDAC were developed [39], which enabled the stratification of patients in risk groups according to survival. The results indicate a biological variation within pancreatic adenocarcinoma that influences patient outcome. These findings are consistent with a study of PDAC tissue [45] describing a methylation signature associated with short survival time and another methylation signature associated with long survival time. Likewise, Minqi Gu et al. [46] identified a four-gene methylation signature (*CCNT1*, *ITGB3*, *SDS* and *HMOX2*) in tumor tissue to predict the prognosis of PDAC patients. Based on the methylation signature patients were divided into a high-risk and a low-risk group [46]. These results indicate that DNA hypermethylation has the potential to provide prognostic information in addition to the TNM classification, which clearly would benefit patients and clinicians’ therapeutic decisions and facilitate the correct choice of treatment.

No doubt, cell-free DNA methylation has prognostic potential as blood-based biomarkers. Nevertheless, the studies in the area are few and validation of the results are lacking. An external validation of the prognostic biomarker studies by our group has just been initiated. Unfortunately, it is still too early to say anything about the outcome. Further research is needed, as well as well-designed prospective studies tracking the methylation profile in cell-free DNA of PDAC patients according to prognosis, treatment and recurrence are.

## 7. Conclusions

The current literature discussed above, clearly indicates that cell-free DNA methylation has the potential to be the fundament of useful blood-based diagnostic and prognostic biomarkers for PDAC, however further research is still needed before clinical application (Table 2).

## Figures and Tables

**Table 1:** Studies on cell-free DNA methylation in plasma/serum as diagnostic biomarkers for pancreatic adenocarcinoma.

Reference	Genes	Method	Cases	Controls	Results	Strenghts	Limitations	Maturity Level *
Nidhi Singh et al., 2020 [33]	*SPARC* *UCHL1* *PENK* *NPTX2*	MSP	PDAC 61	CP 22 HC 21	*SPARC:* AUC of 0.76 (0.65–0.87) The other genes did not reach statistical significance.	- Consecutive inclusion of PDAC patients. - Large group of well-defined cases. - Inclusion of controls with benign pancreatic disease.	- Lack information on whether cases and controls were matched according to age, sex etc. - No information on treatment before blood sampling.	Phase 1/Phase 2. No further validation.
Fujimoto et al., 2020 [36]	*RUNX3*	CORD assay	PDAC 55	CP 12 HC 80	*RUNX3:* Sens; 50.9%, Spec; 93.5% (the total control group) Combining *RUNX3* and CA-19-9: Sens; 85.5%, Spec; 93,5%	- Large group of cases and controls. - Inclusion of controls with benign pancreatic disease.	- Not matched according to age and sex. - No information on treatment before blood sampling.	Phase 1/Phase 2. No validation studies.
Shinjo et al., 2020 [37]	*ADAMTS1* *HOXA1* *PCDH10* *SEMA5A* *SPSB4*	qMSP	PDAC 47	HC 14	The panel of five genes (with at least one gene methylated): Sens; 49%, Spec; 86% Combining the gene panel and *KRAS* mutation in cell-free DNA: Sens; 68%, Spec; 86%	- Well described and comprehensive analysis of tissue samples. - Pretreatment blood samples. - Cases and controls matched according to age and sex.	- Lacking a control group of patients with benign pancreatic disease.	Phase 1/Phase 2. No validation studies
Henriksen et al., 2017 [25]	*SFRP2* *SFRP1* *APC* *TFPI2* *MESTv2* *BNC1* *RASSF1A* *BMP3*	MSP	PDAC 95	CP 97 SN 27	Prediction model combining hypermethylation status of the eight genes and age <65 years: PDAC vs. CP and SN: AUC 0.86 (0.81–0.91)	- Consecutive inclusion of PDAC patients. - Large group of cases and controls with benign pancreatic disease. - Pretreatment plasma	- Cases and controls not age-matched. - The gene-panel is not analyzed in tumor tissue.	Phase 1/Phase 2. An external validation study is initiated.
Eissa et al., 2019 [32]	*BNC1* *ADAMTS1*	MOB and qMSP	PDAC 39	HC 95 CP 8	Combining *BNC1* and *ADAMTS1*: PDAC vs HC: AUC 0.95 (0.91–0.98) CP: 7/8 were had either *BNC1* or *ADAMTS1* methylated.	- Population-based matched and age-matched controls. - Pretreatment serum.	- Small control group of CP patients - Racial difference between cases and controls.	Phase 1/Phase 2. Validation study of Joo Mi Yi et al., 2013 [31].
Joo Mi Yi et al., 2013 [31]	*BNC1* *ADAMTS1*	MOB	PDAC 42	HC 26	*BNC1:* PDAC 79% (33/42), HC 11,5% (3/26) *ADAMTS1:* PDAC 48% (20/42), HC 7.7% (2/26) Combining *BNC1* and *ADAMTS1* did not improve sensitivity and specificity.	- Well described and comprehensive analysis of cell-lines and tissue samples. - Pretreatment serum.	- Lack information on whether cases and controls were matched according to age, sex etc. - Lacking a control group of patients with benign pancreatic disease	Phase 1 and phase 2. One validation study is performed (Eissa et al., 2019 [32]).
Kawasaki et al., 2013 [30]	*APC* *DCC* *P16* *P14* *RASSF1A*	MSP	PDAC 47	Patients with other types of cancer 197	*APC:* 23.4% (11/47) *DCC:* 6.4% (3/47) *P16:* 17% (8/47) *P14:* 14.9% (7/47) *RASSF1A:* 34% (16/47)	- Pretreatment plasma. - Large cohort of cases.	- Lack of HC or controls of patients with benign pancreatic disease.	Phase 1/Phase 2. No validation studies.
Park, Ryu et al., 2012 [29]	*NPTX2*	qMSP	PDAC 104	CP 60 GD 5	Frequency of hypermethylation: PDAC: 84% (87/104) CP: 33% (20/60) GD: 0% (0/5) Statistically significant difference (*P* = 0.016)	- Pretreatment plasma. - Large cohort of cases and controls. - Include controls with benign pancreatic disease.	- Not age matched cases and controls - No description of inclusion period.	Phase 1/Phase 2. A validation study of NPTX2 (Park, Baek et al., 2012 [28]). No further validation.
Park, Baek et al., 2012 [28]	*NPTX2* *UCHL1* *SARP2* *ppENK* *P16* *RASSF1A*	MSP	PDAC 16	CP 13 HC 29	Frequency of hypermethylation: *NPTX2:* PDAC 6/16, CP 2/13, HC 0/29 *UCHL1:* PDAC 4/16, CP 2/13, HC 0/29 *SARP2:* PDAC 5/16, CP 3/13, HC 0/29 *ppENK:* PDAC 5/16, CP 2/13, HC 0/29 *P16:* PDAC 4/16, CP 2/13, HC 1/29 *RASSF1A:* PDAC 1/16, CP 1/13, HC 0/29 No statistically significant difference.	- Include controls with benign pancreatic disease.	- Small cohort of cases and controls. - No information on treatment before blood sampling. - Not age-matched cases and controls.	Phase 1/Phase 2. NPTX2 was subsequently validated (Park, Ryu et al., 2012 [29]). No further validation.
Liggett et al., 2010 [27]		MethDet56	PDAC 30	CP 30 HC 30	- 8 gene promoters could differentiate CP and HC: Sens; 78.0%, Spec; 81.7% - 14 gene promoters could differentiate PDAC and CP: Sens; 90.8%, Spec; 91.2	- Cases and controls matched on sex, age and race. - Detailed description of CP patients. - Pretreatment plasma.	- No information on whether the aberrantly methylated genes were hypo- or hypermethylated. - No description of inclusion period **.	Phase 1/Phase 2. No validation studies.
Melnikov et al., 2009 [26]		MethDet56	PDAC 30	CP 30 HC 30	Based on five hypomethylated promoter regions: Sens; 76%, Spec; 59%	- Cases and controls matched on sex and age.	- No description of inclusion period **. - No information on treatment before blood sampling.	Phase 1/Phase 2. No validation studies.

PDAC; Pancreatic ductal adenocarcinoma, CP; Chronic pancreatitis, SN; Patients with symptoms mimicking upper gastrointestinal cancer, but not having cancer, HC; Healthy controls, GD; Gallstone disease; Sens; Sensitivity. Spec; Specificity; MethDet56; Microarray-mediated methylation analysis of 56 fragments, MSP; Methylation specific PCR, qMSP; Quantitative methylation specific PCR; * Maturity level according to phases of biomarker development defined by Pepe et al, 2001 [38]. Phase 1: Preclinical explorative studies – is often performed on tumor tissue and non-tumor tissue to identify a promising direction for a potential useful biomarker. Phase 2: Clinical assay and validation studies—is performed on a non-invasive specimen e.g., plasma or serum to detect an established disease and distinguish cancer subjects from subjects without cancer. Phase 3: Retrospective longitudinal studies—the biomarker detects disease early before it becomes clinical e.g., clinical specimens collected from cancer subjects before their clinical diagnosis of cancer are compared to specimens from controls who have not developed cancer. Phase 4: Prospective screening studies—the performance (both the detection rate and the false referral rate) of the biomarker is identified in a relevant population. Phase 5: Cancer control studies—is to estimate the reduction in cancer mortality afforded by screening with the biomarker. ** Both studies included patients from the same center, as none of the studies describe the inclusion period, it is impossible to exclude patient overlap.

**Table 2:** Summary of Recommended Future Directions for the Field.

• Cases and controls matched according to age, sex and race.
• The use of pretreatment blood samples.
• Studies combining methylated gene-panels and other diagnostic modalities.
• Large validation studies including cases with early stage cancer and controls with benign pancreatic disease.
• Validation on cases with pancreatic cancer precursor lesions.
• Validation on cases with increased risk of pancreatic cancer e.g., subjects with a risk of familial pancreatic cancer and subjects with diabetes.
• Retrospective longitudinal studies.
• Prospective screening studies of subjects with increased risk of pancreatic cancer.

## Abbreviations

Pancreatic adenocarcinoma	(PDAC)
Carbohydrate antigen-19-9	(CA-19-9)
List of Genes:	
*ADAMTS1*	A disintegrin and metalloproteinase with thrombospondin motifs 1
*APC*	Adenomatous polyposis coli
*ALX4*	Homebox Protein Aristaless-Like 4
*BNC1*	Basonuclin 1
*BMP3*	Bone Morphogenetic Protein 3
*CCNT1*	Cyclin T1
*CDKN2A*	Cycklin-Dependent Kinase Inhibitor 2A (p16)
*CDKN2B*	Cycklin-Dependent Kinase Inhibitor 2B (p15)
*DCC*	Deleted in Colorectal Carcinoma
*HMOX2*	Heme Oxygenase 2
*HOXA1*	Homeobox protein Hox A1
*HIC1*	Hypermethylated in Cancer 1
*ITGB3*	Integrin beta-3
*MESTv2*	Mesoderm Specific Transcript 2
*MLH1*	MutL Homolog 1
*NEUROG1*	Neurogenin 1
*NPTX2*	Neuronal pentraxin 2
*ppENK*	Preproenkephalin
*PCDH10*	Protocadherin 10
*RASSF1A*	Rras associated domain family member 1
*RUNX3*	Runt-related transcription factor 3
*SARP1*	Secreted apoptose related protein 1
*SARP2*	Secreted apoptose related protein 2
*SPARC*	Secreted Protein Acidic and Cysteine Rich (SPARC)
*SEMA5A*	Semaphorin 5A
*SEPT9v2*	Septin 9 Transcript Variant 2
*SDS*	Serine Dehydratase
*SST*	Somatostatin
*SPSB4*	SplA/Ryanodine Receptor Domain and SOCS Box Containing 4
*TAC1*	Tachykinin Precusor 1
*TFPI2*	Tissue factor pathway inhibitor 2
*UCHL1*	Ubiquitin carboxy-terminal hydrolase L1
*WNT5A*	Wingless-Type MMTV Integration Site Family, Member 5A
